# Validity and Reliability of 10-Hz Global Positioning System to Assess In-line Movement and Change of Direction

**DOI:** 10.3389/fphys.2018.00228

**Published:** 2018-03-15

**Authors:** Pantelis T. Nikolaidis, Filipe M. Clemente, Cornelis M. I. van der Linden, Thomas Rosemann, Beat Knechtle

**Affiliations:** ^1^Exercise Physiology Laboratory, Nikaia, Greece; ^2^Instituto Politécnico de Viana do Castelo, Escola Superior de Desporto e Lazer, Viana do Castelo, Portugal; ^3^Instituto de Telecomunicações, Lisbon, Portugal; ^4^JOHAN Sports, Department of Sport Sciences, Noordwijk, Netherlands; ^5^Institute of Primary Care, University of Zurich, Zurich, Switzerland; ^6^Mebase St. Gallen Am Vadianplatz, St. Gallen, Switzerland

**Keywords:** GPS, team sport, tracking, direction, change

## Abstract

The objectives of the present study were to examine the validity and reliability of the 10 Hz Johan GPS unit in assessing in-line movement and change of direction. The validity was tested against the criterion measure of 200 m track-and-field (track-and-field athletes, *n* = 8) and 20 m shuttle run endurance test (female soccer players, *n* = 20). Intra-unit and inter-unit reliability was tested by intra-class correlation coefficient (ICC) and coefficient of variation (CV), respectively. An analysis of variance examined differences between the GPS measurement and five laps of 200 m at 15 km/h, and *t*-test examined differences between the GPS measurement and 20 m shuttle run endurance test. The difference between the GPS measurement and 200 m distance ranged from −0.13 ± 3.94 m (95% CI −3.42; 3.17) in the first lap to 2.13 ± 2.64 m (95% CI −0.08; 4.33) in the fifth lap. A good intra-unit reliability was observed in 200 m (ICC = 0.833, 95% CI 0.535; 0.962). Inter-unit CV ranged from 1.31% (fifth lap) to 2.20% (third lap). The difference between the GPS measurement and 20 m shuttle run endurance test ranged from 0.33 ± 4.16 m (95% CI −10.01; 10.68) in 11.5 km/h to 9.00 ± 5.30 m (95% CI 6.44; 11.56) in 8.0 km/h. A moderate intra-unit reliability was shown in the second and third stage of the 20 m shuttle run endurance test (ICC = 0.718, 95% CI 0.222;0.898) and good reliability in the fifth, sixth, seventh and eighth (ICC = 0.831, 95% CI −0.229;0.996). Inter-unit CV ranged from 2.08% (11.5 km/h) to 3.92% (8.5 km/h). Based on these findings, it was concluded that the 10 Hz Johan system offers an affordable valid and reliable tool for coaches and fitness trainers to monitor training and performance.

## Introduction

A global positioning system (GPS) is a satellite-based navigational technology that has been used extensively in outdoor team sports to track the players' activity (Cummins et al., 2013). Small portable GPS units have been progressively used to quantify players' locomotion and to characterize the external load (work performed) of training sessions and matches (Portas et al., 2010; Bourdon et al., 2017). Based on the information of GPS technology, it is possible to measure basic components of players' patterns of movement, speed, distance covered and accelerations/decelerations in combination with inertial measurement unit, thus characterizing the physical impact of the session and evaluating the training programs (Cummins et al., 2013; Malone et al., 2017). Such metrics can be used in real-time or post data processing to control the training impact and to adjust the stimulus to find the “sweet-spot” of progressive training load and avoid injury risk situations (Gabbett, 2016).

Despite of the practical applications of this technology, some issues have been discussed (Malone et al., 2017): (i) reliability and validity of the device; (ii) data collection and processing; (iii) satellite connection and horizontal dilution of precision; and (iv) data exclusion criteria. GPS trackers are often commercialized and used before essential independent information about the precision and accuracy of the data is known (Russell et al., 2016). Both validation and accuracy are important contributors to ensure the quality of the information, thus essential independent studies allow confirmation of the usability of the data (Vickery et al., 2014). GPS devices are currently manufactured with 5- and 10-Hz sampling rates, suggesting that higher frequency rates provide greater validity for measuring distance (Cummins et al., 2013). Usually, GPS trackers are validated by using a tape measure to measure the distance between the timing gates at the start and finish to compare speed (Waldron et al., 2011). Comparisons with other tracking technologies such as a semi-automatic system or local position measurement have been also conducted (Buchheit et al., 2014; Beato et al., 2016). Frequency rates of 5-Hz seem to be enough to guarantee an acceptable level of accuracy and reliability for total distance (~10% of variance) (Coutts and Duffield, 2010), although not satisfactory to measure high-speed running (Rampinini et al., 2015) or rapid directional change (Rawstorn et al., 2014). Based on that, 10-Hz units or higher combined with an inertial measurement unit (>100-Hz) have now been recommended to ensure the necessary level of accuracy and precision (Aughey, 2011; Rampinini et al., 2015).

Validation of GPS devices is usually done by completing a standard circuit, running at a linear sprint or with changes of direction, and uses specific tasks that simulate the game (Beato et al., 2016). In most cases, the validation studies only focus on one specific analysis (total distance or high-speed running), one kind of task (circuit, linear sprint or change of direction) and one type of comparison (tape measure, timing gates or other tracking methods) (Coutts and Duffield, 2010; Portas et al., 2010; Buchheit et al., 2014; Vickery et al., 2014). However, there is limited research that uses an integrative approach with multiple analyses, kinds of tasks and types of comparisons to test the validity and reliability of GPS units. Based on that, the purpose of this article was to determine the validity and reliability of the 10-Hz JOHAN sports tracker during straight line running and multi-direction movement patterns by comparing with a tape measure.

## Methods

The present cross-sectional study included two parts; in the first part, participants (female, *n* = 6, and male, *n* = 2, track-and-field athletes; age 13.1 ± 1.1 years, weight 49.9 ± 5.8 kg, height 163 ± 8 cm) performed five 200-m runs across a 200-m track-and-field stadium, whereas in the second part, participants (female soccer players, *n* = 20, age 15.5 ± 2.7 years, weight 60.9 ± 9.5 kg, height 162 ± 4 cm) performed the 20-m shuttle run endurance test. All participants' parents or guardians provided consent after having been informed about the content of the study. The study design was approved by the local institutional review board (Ethics Committee, Exercise Physiology Laboratory, Nikaia, Greece). In the first study, participants were eight young track-and-field athletes who performed five laps of 200 m high-intensity running (~48 s per lap, 15 km/h) with a 1 min break wearing the Johan GPS (JOHAN Sports, Noordwijk, Netherlands) consisting of a GPS sensor (10 Hz, including EGNOS correction), accelerometer, gyroscope and magnetometer (100 Hz, 3 axis, ±16 g). In the second study, participants were 20 female soccer players, members of a club participating in the first national league. All participants received the motion trackers before the warm-up to become familiarized with them. The motion trackers were worn in a body tight vest between the scapulae.

In the first study, participants were instructed to start the 200-m runs from a standstill and to slow their speed immediately at the finish. They ran in a single group consisting of four pairs and were asked to be close to each other continuously. The 200 m runs were captured separately and were repeated for each participant. The start of the 200 m run was chosen when the speed started to increase exponentially, whereas the end of 200 m run was highlighted after the speed started to decrease. In the 20 m shuttle run test, participants were instructed to run between two lines 20 m apart at a pace dictated by audio signal. The test started at 8.0 km/h with the speed increasing by 0.5 km/h every minute. It finished when the participants either stopped due to fatigue or failed to follow the pace on two consecutive occasions (Vanhelst et al., 2017). The number of shuttles (20 m) varies as the test progresses, e.g., seven shuttles (i.e., 140 m) are performed at 8.0 km/h and eight shuttles at 8.5 km/h. There were light clouds during the two testing days and there were no high buildings in the surroundings. Motion data from the trackers were uploaded post-experimentally to the JOHAN Sports online analysis platform. For both studies the JOHAN Software was used to capture the 200-m runs and shuttle runs motion data. This capturing was executed using 1 s data resolution (aggregated from 10 Hz motion data). The capturing was executed by one person who had three years of experience working with JOHAN Software. In the second study, participants ran multiple sets of shuttles with different speeds in the context of the 20 m shuttle run test. The capturing of the 200 m runs and 20 m shuttles were carried out for each player, separately. The start of one set of shuttles was chosen when the speed started to increase exponentially, whereas the end of one set of shuttles was highlighted by the dip in the speed (before the next set of shuttles started). Finally, all the capturing was exported from JOHAN to Excel for statistical analyses.

### Statistical analyses

All statistical analyses were performed using SPSS and Graphpad. The validity was tested against the gold standard of real distance (200 and 20 m with change of direction in the first and second study, respectively). An athletic track was also previously used as the criterion measure in the validation of a GPS system (Petersen et al., 2009). A repeated measures analysis of variance (ANOVA) examined differences between GPS measurements and five laps of 200 m at 15 km/h. The magnitude of these differences was examined using eta squared (η^2^) and evaluated as: small (0.010 < η^2^ ≤ 0.059), moderate (0.059 < η^2^ ≤ 0.138) and large (η^2^ > 0.138) (Cohen, 1988). The paired samples *t*-test examined differences between GPS measurements and 20 m shuttle run endurance test. The magnitude of the differences in the *t*-test was determined using the following criteria of Cohen's d: *d* ≤ 0.2, trivial; 0.2 < *d* ≤ 0.6, small; 0.6 < *d* ≤ 1.2, moderate; 1.2 < *d* ≤ 2.0, large; and *d* > 2.0, very large (Batterham and Hopkins, 2006). Validity was assessed using the standard error of the estimate (SEE), which was calculated as the SD (±90% CI) of the % difference between the known distance and the GPS recorded distance for each trial (Jennings et al., 2010). The percentage difference between the known distance and the GPS recorded distance was also calculated to indicate bias (Petersen et al., 2009). The percentage difference between the GPS recorded and the known distance was calculated as 100^*^(GPS recorded distance-known distance)/known distance. In addition, the GPS recorded distance and the known distance were compared using Bland-Altman plot, where the difference was calculated as recorded minus known distance and the average as (recorded-known distance)/2. Intra-class correlation coefficient (ICC) tested intra-unit reliability among exercises of the same distance, i.e. in study 1, among the five laps, and in study 2, between stages of 160 m (8.5 and 9.0 km/h) and among stages of 200 m (10, 10.5, 11.0, and 11.5 km/h). ICC was interpreted as poor (<0.5), moderate (0.5–0.75), good 0.75 and 0.90, and excellent (>0.90). Inter-unit reliability was tested using coefficient of variation (CV) considering the performance of the same movements by different participants (Duffield et al., 2010). Statistical significance for all calculations was set at alpha = 0.05.

## Results

### Study 1

**Figure 1:**
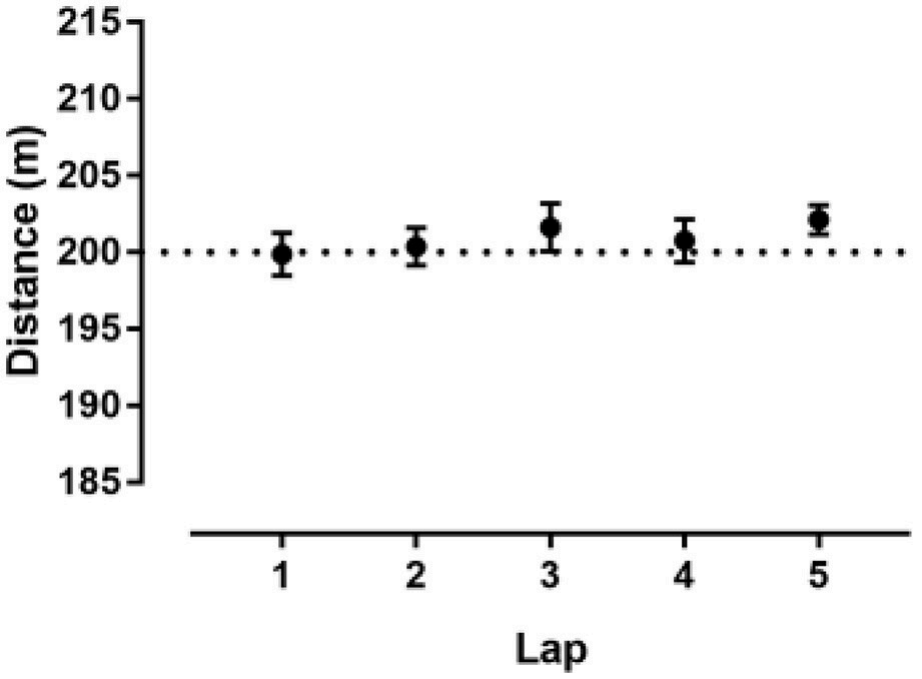
Estimated distance by GPS of 200 m. Error bars represent standard error of measure. The dashed line represents 200 m distance.

**Figure 2:**
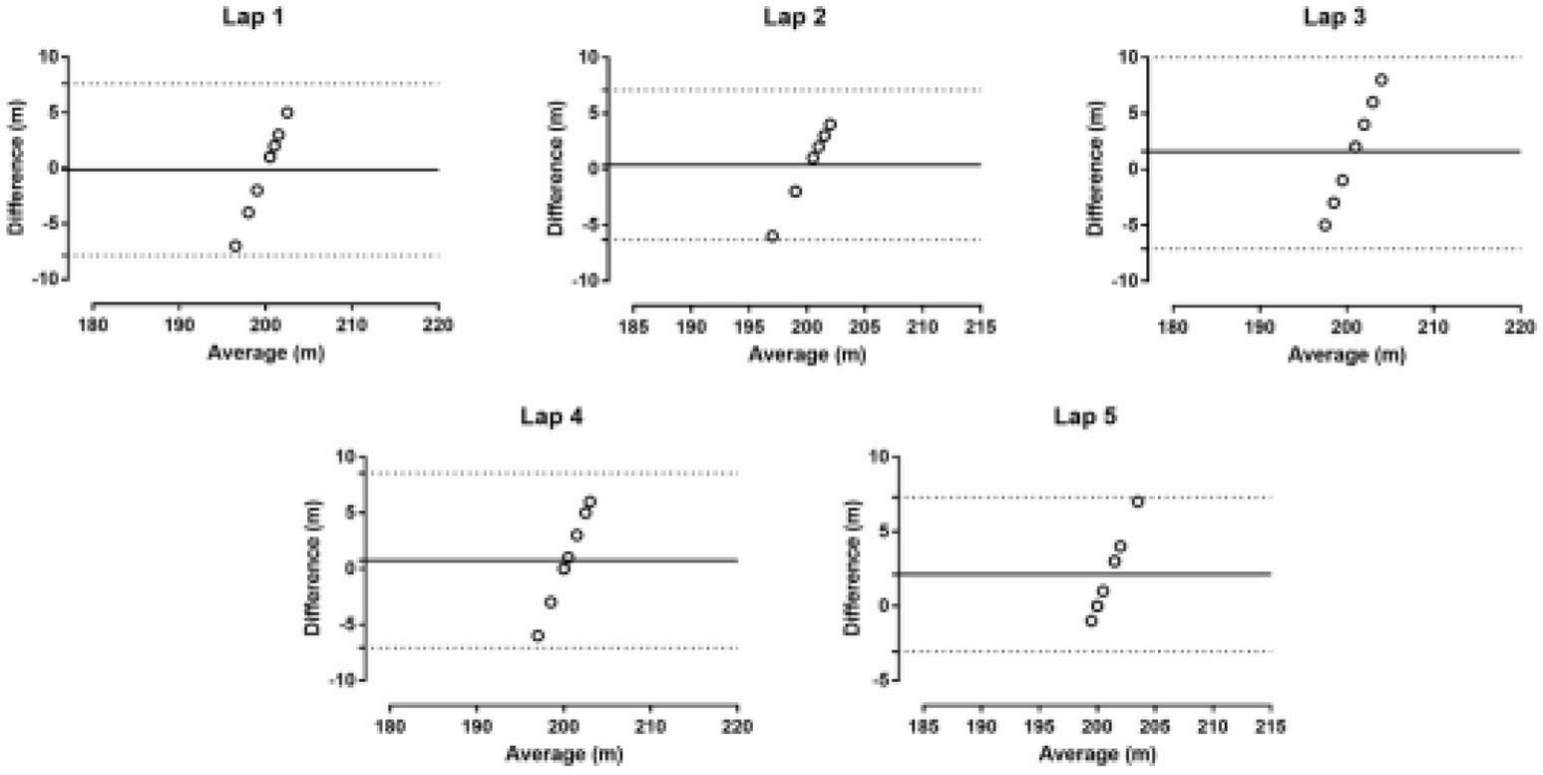
Bland-Altman plots in five laps of 200 m. The solid line represents the bias. The dashed lines represent 95% limits of agreement.

No statistically significant difference was observed among the five 200 m GPS recorded distance trials and the known 200 m distance (*p* = 0.436, η^2^ = 0.119). The difference between GPS measure and 200 m distance was −0.13 ± 3.94 m (95% CI −3.42; 3.17) in the first, 0.38 ± 3.42 m (95% CI −2.48; 3.23) in the second, 1.63 ± 4.44 m (95% CI−2.09; 5.34) in the third, 0.75 ± 3.99 m (95% CI −2.59; 4.09) in the fourth and 2.13 ± 2.64 m (95% CI −0.08; 4.33) in the fifth lap (Figure 1, Table 1). The mean difference between the GPS recorded distance and the reference distance was less than ~1%. The Bland-Altman plot for each lap is shown in Figure 2. A good intra-unit reliability was observed at 200 m (ICC = 0.833, 95% CI 0.535; 0.962). Inter-unit CV ranged from 1.31% (fifth lap) to 2.20% (third lap) (Table 1).

**Table 1:** GPS recorded distance for each participant in the five laps of 200 m.

**Participants**	**Lap**				
	**1**	**2**	**3**	**4**	**5**
1	202	203	208	201	207
2	196	198	202	194	199
3	201	204	202	203	201
4	193	194	197	200	200
5	201	198	204	197	200
6	198	201	195	200	203
7	203	202	199	206	203
8	205	203	206	205	204
Mean difference (%)	−0.06	0.19	0.81	0.38	1.06
SD	0.70	0.60	0.78	0.71	0.47
90% CI	−1.38;1.26	−0.96;1.33	−0.67;2.30	−0.96;1.71	0.18;1.95
CV (%)	1.97	1.71	2.20	1.99	1.31

*CV, coefficient of variation*.

### Study 2

**Figure 3:**
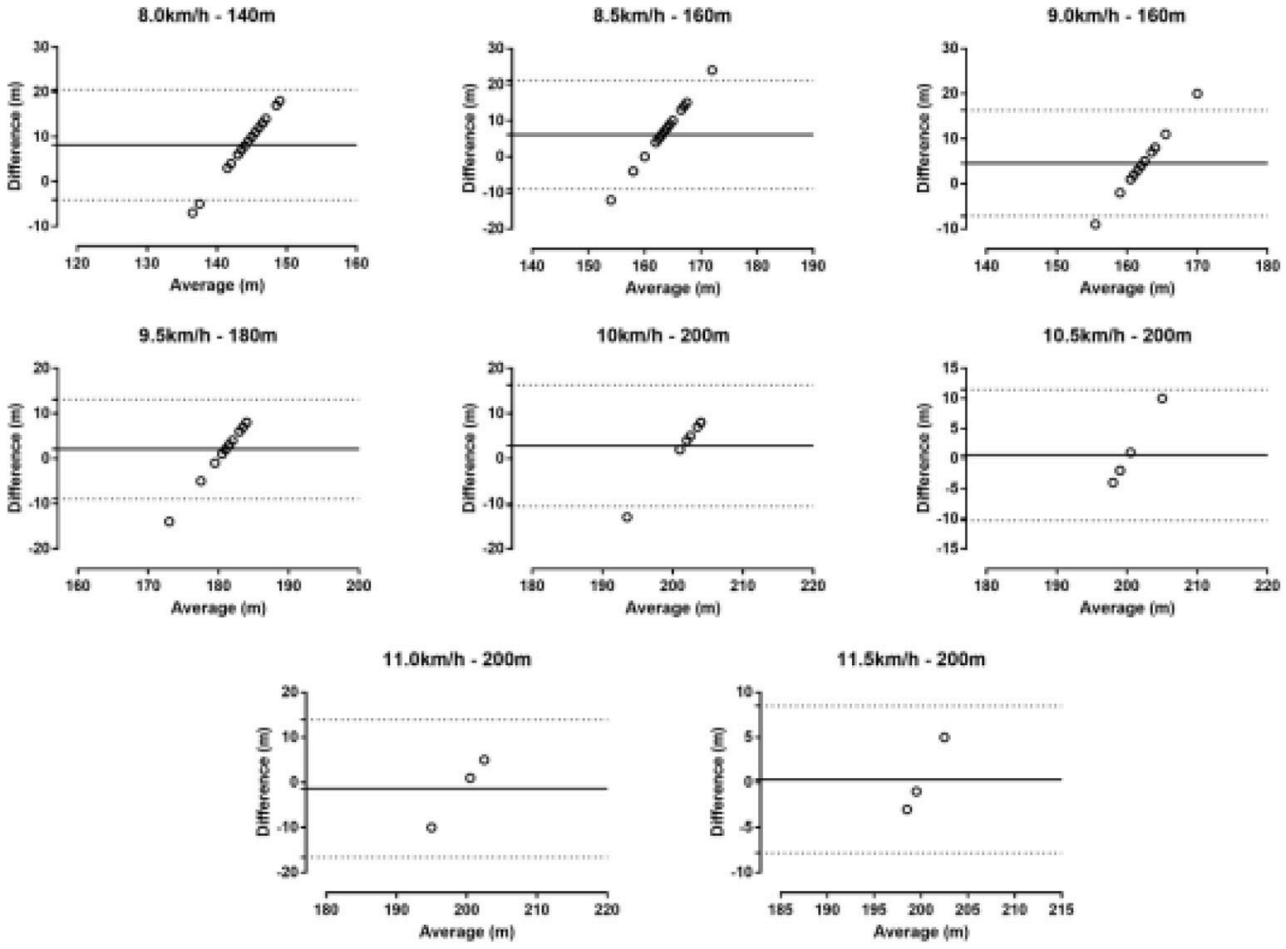
Bland-Altman plots in the 20 m shuttle run test. The solid line represents the bias. The dashed lines represent 95% limits of agreement.

A statistically significant difference was observed between the GPS recorded distance and the known distance at 8.0 km/h (*p* < 0.001, *d* = 1.85), 8.5 km/h (*p* = 0.002, *d* = 1.13) and 9 km/h (*p* = 0.006, *d* = 1.09), but not at 9.5 km/h (*p* = 0.167, *d* = 0.53), 10.0 km/h (*p* = 0.274, *d* = 0.59), 10.5 km/h (*p* = 0.821, *d* = 0.15), 11.0 km/h (*p* = 0.794, *d* = −0.24) and 11.5 km/h (*p* = 0.902, *d* = 0.11). The difference between GPS measure and 20 m shuttle run endurance test was 9.00 ± 5.30 m (95% CI 6.44; 11.56) at 8.0 km/h, 7.11 ± 6.55 m (95% CI 3.95; 10.26) at 8.5 km/h, 4.59 ± 5.98 m (95% CI 1.51; 7.66) at 9.0 km/h, 2.13 ± 5.67 m (95% CI −1.01; 5.27) at 9.5 km/h, 1.20 ± 8.23 m (95% CI −9.02; 11.42) at 10.0 km/h, 0.60 ± 5.55 m (95% CI −6.29; 7.49) at 10.5 km/h, −1.33 ± 7.77 m (95% CI −20.63; 17.96) at 11.0 km/h and 0.33 ± 4.16 m (95% CI −10.01; 10.68) at 11.5 km/h (Table 2). The mean difference between the GPS recorded distance and the reference distance was less than ~5%. The Bland-Altman plot for each lap is shown in Figure 3. A moderate intra-unit reliability was shown in the second and third stage of the 20 m shuttle run endurance test (ICC = 0.718, 95% CI 0.222;0.898) and good reliability in the fifth, sixth, seventh, and eighth (ICC = 0.831, 95% CI −0.229;0.996). Inter-unit CV ranged from 2.08% (11.5 km/h) to 3.92% (8.5 km/h) (Table 2).

**Table 2:** GPS recorded distance for each participant in the 20-m endurance shuttle run test.

	**Speed (km/h)**								
	**8.0**	**8.5**	**9.0**	**9.5**	**10.0**	**10.5**	**11.0**	**11.5**	**12.0**
	Distance (m)^*^								
	**140**	**160**	**160**	**180**	**200**	**200**	**200**	**200**	**220**
**PARTICIPANTS**									
1	146	160	164	186	204	210	205	205	199
2	146	156	161	175	187	196	190	199	
3	154	167	165	186	208	198	201	197	
4	157	175	168	188	205	198			
5	143	156	162	179	202	201			
6	147	164	163	181	207				
7	150	168	167	187	202				
8	151	173	161	182	208				
9	149	164	158	184					
10	158	174	164	183					
11	146	184	180	188					
12	153	165	171	183					
13	149	166	168	182					
14	148	170	165	182					
15	135	167	165	166					
16	150	169	165						
17	152	165	151						
18	153	166							
19	144	166							
20	133	148							
Mean difference (%)	5.37	4.04	2.67	1.08	1.31	0.24	−0.78	0.14	
SD	4.18	5.27	3.52	3.24	3.53	2.69	4.01	2.06	
90% CI	3.75;6.98	2.00;6.08	1.18;4.16	−0.40;2.55	−1.05;3.68	−2.32;2.80	−7.53;5.98	−3.33;3.61	
CV (%)	3.56	3.92	3.63	3.11	3.38	2.77	3.91	2.08	

* *Distance covered in each speed varies due to the different number of shuttles performed. CV, inter-unit coefficient of variation*.

## Discussion

The main findings of the present study were that Johan GPS system (i) accurately measured the distance in the 200 m and in the relatively fast stages of the 20 m shuttle run test; (ii) had inter-unit CV lower than 3.92% at short distances and 2.20% at longer distances; and (iii) had moderate-to-good intra-unit reliability in short and long distances, and the reliability was larger at relatively faster speeds. These results suggest that 10-Hz JOHAN sports GPS is valid and reliable for linear movements typically observed in team sports such as soccer. However, these properties differed between running long and short distances.

We examined the validity of the Johan GPS system against the gold standard of real distance (Muñoz-Lopez et al., 2017). Overall, the GPS shows accurate values since no difference was observed between measured and real distance in 200 m and in the relatively fast speeds of the 20 m shuttle run test. On the other side, the GPS overestimated the distance in the low speeds of the test, which should be attributed to the participants' behavior. Particularly, the participants might perform excess movements in the change of direction during the first slow stages of the test, whereas, as the test proceeded, they became more careful in order to avoid unnecessary movements that would result in additional fatigue. The ability of successful change of direction is related to speed, reactive strength, power and balance (Sheppard and Young, 2006) and characterizes athletes of team sports such as soccer (St Clair Gibson et al., 1998). Although the soccer players participating in the present study were experienced and were accustomed to the 20 m shuttle run test from previous testing sessions, the excess movements in the first levels of the test might partially explain the smaller accuracy of the GPS in this part of the test.

With regards to the reliability of the GPS, a previous review on acceptable error in GPS suggested CV values <5% can be classified as good, 5.1–10% moderate and greater than 10% poor results (Scott et al., 2016). In study 1, the inter-unit CV ranged from 1.31% (fifth lap) to 2.20% (third lap) and in study 2, inter-unit CV ranged from 2.08% (11.5 km/h) to 3.92% (8.5 km/h), thus suggesting that the 10-Hz GPS (Johan Sports) ensures good results and can be classified as reliable to measure both long and short distances. The lower inter-unit reliability in the shorter distance might be due to the effect of acceleration and the change of direction. Previous research has shown that the validity of 10 Hz GPS is inversely related to acceleration (Akenhead et al., 2014). Moreover, it has been observed that fast change of direction reduces the accuracy of GPS (Rawstorn et al., 2014). For instance, a comparison of linear and non-linear 200 m courses showed larger error in the latter (Gray et al., 2010).

Ten Hz GPSs are more valid than GPS units with smaller sampling frequency such as 1 Hz (Coutts and Duffield, 2010) or 5 Hz (Duffield et al., 2010). A comparison between 1 and 5 Hz showed that a higher frequency rate improved validity (Jennings et al., 2010). A 10 Hz unit has been proved three times more valid and six times more reliable than 5 Hz unit (Varley et al., 2012). However, a comparative study of 10 and 15 Hz showed higher validity in the former than in the latter (Johnston et al., 2014). An explanation of the improved validity of GPS with increased sampling frequency might be that the larger sampling frequency results in the theoretically more precise identification of motion. For instance, a 10 Hz unit can analyze a motion with precision 0.1 s, whereas a 5 Hz unit can analyze with 0.2 s precision.

A limitation of this study was that it focused on linear movements of moderate intensity; thus, the findings should be generalized with caution to other modes of movements (such as multi-directional) and different speeds. One strength of this study is that it included 20 m with change of direction as well as linear running, and both are relevant for soccer. Considering the wide use of GPS units to monitor training and performance in team sports (Aughey and Falloon, 2010; Castellano and Casamichana, 2010; Wisbey et al., 2010; Clemente et al., 2017), the results of the present study will help coaches and trainers optimize their work. The results are of great practical value for professionals (e.g., coaches, fitness trainers, exercise physiologists, analysts) working with team sport players, especially soccer, as they demonstrate that a 10-Hz GPS system is a valid and reliable tool to monitor training. The error found by the GPS unit can be used by soccer professionals for detecting changes in performance (Waldron et al., 2011). Furthermore, this particular model offers an inexpensive solution compared to other commercially available models. Future studies should examine the validity and reliability of this GPS unit in larger samples of athletes performing more sport-specific movements.

## Conclusion

Based on the findings of the present study, we conclude that the 10-Hz Johan GPS system is a valid and reliable tool that professionals working with team sport players and endurance runners can use to monitor training involving linear in-line movement and change of direction. Moreover, those using this equipment should be aware of the differences in its accuracy between monitoring long-distances and short distances with change of direction.

## Author contributions

FC: Writing paper; CvdL: Data analysis; PN: Data collection and drafting paper; TR: Writing paper; BK: Writing paper.

### Conflict of interest statement

The authors declare that the research was conducted in the absence of any commercial or financial relationships that could be construed as a potential conflict of interest.
